# Emerging trends in prime editing for precision genome editing

**DOI:** 10.1038/s12276-025-01463-8

**Published:** 2025-07-31

**Authors:** Jaesuk Lee, Jiyeon Kweon, Yongsub Kim

**Affiliations:** 1 nSAGE Inc., Incheon, Republic of Korea; 2 Department of Cell and Genetic Engineering, BK21 Project, Asan Medical Institute of Convergence Science and Technology, Asan Medical Center, University of Ulsan College of Medicine, Seoul, Republic of Korea; 3 Stem Cell Immunomodulation Research Center, University of Ulsan College of Medicine, Seoul, Republic of Korea

**Keywords:** Genetic engineering, Gene targeting, Targeted gene repair

## Abstract

Prime editing is an advanced genome editing technology that enables precise genetic modifications without inducing double-strand breaks or requiring donor DNA templates. Prime editing has rapidly become a versatile tool, supporting a wide range of genetic modifications, including point mutations, insertions and deletions. Here we examine the evolution of prime editing technologies, detailing advancements from the initial prime editing systems to recent innovations that enhance editing efficiency. Through structural modifications and improved delivery methods, prime editing has expanded its applicability across eukaryotic systems. By enabling access to previously challenging mutations, prime editing opens new avenues for therapeutic development and precision genetic research, with efficiency, specificity and accessibility expected to shape its future impact in genome engineering.

## Prime editing revolutionizes genetic modifications in research

This Review discusses advancements in gene-editing technologies, focusing on prime editing. Traditional CRISPR systems cause double-strand breaks in DNA, leading to unintended mutations, while base editors often induce unwanted bystander edits and are restricted to specific base conversions. Prime editing offers a more precise alternative by avoiding double-strand breaks and allowing targeted genetic changes. Using a modified Cas9 enzyme and specialized guide RNAs, prime editing can efficiently perform all types of base conversions, small insertions and deletions. Researchers have developed several versions of prime editors, each improving efficiency and reducing errors. The study highlights the potential of prime editing in various fields, including medicine and agriculture. It can correct genetic mutations linked to diseases and improve crop traits. However, challenges remain in delivering these tools effectively to cells and ensuring high precision.

This summary was initially drafted using artificial intelligence, then revised and fact-checked by the author.

## Introduction

The study of pathogenic mutations found in patients and the development of gene therapies to address these genetic alterations have garnered substantial attention in recent years. Gene-editing technologies, such as zinc finger nucleases, transcription activator-like effector nucleases and the CRISPR–Cas9 nuclease, have emerged as powerful tools, enabling precise modification of the genome by targeting specific loci with high fidelity^1,2^. These nucleases have become indispensable in various fields, ranging from functional genomics to potential therapeutic applications^3,4^. However, despite their success, these approaches have limitations, such as inducing double-strand breaks (DSBs), which can activate p53, leading to cellular stress, apoptosis and unpredictable repair outcomes such as insertions, deletions and chromosomal rearrangements^5–10^. These drawbacks underscore the need for more precise and safer gene-editing modalities.

**Fig. 1:**
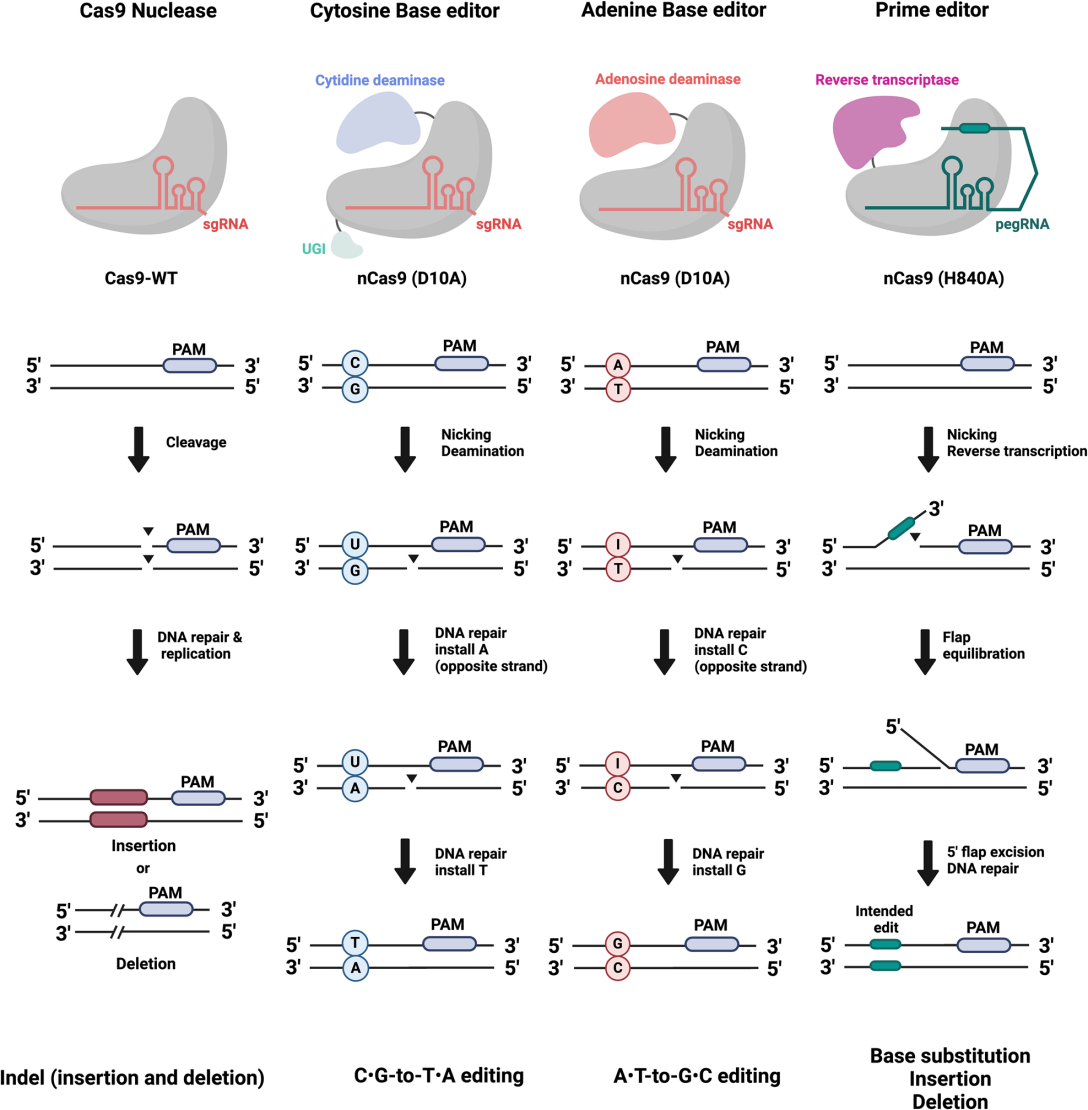
Overview of genome editors and editing outcomes. A Cas9 nuclease, cytosine BE (CBE), adenine BE (ABE) and prime editor (PE) are shown, illustrating their mechanisms of action and respective editing outcomes. The Cas9 nuclease (Cas9-WT), guided by a sgRNA, induces a DSB at the target site, leading to DNA repair via nonhomologous end joining (NHEJ) or HDR, resulting in indels. CBE combines nCas9 (D10A) and cytidine deaminase to convert a cytosine (C) to uracil (U), which is repaired as a thymine (T), achieving C-to-T substitutions. The ABE uses nCas9 (D10A) fused with adenosine deaminase to convert adenine (A) to inosine (I), which functions as guanine (G), resulting in A-to-G substitutions. The prime editor (PE) is a more versatile system, using nCas9 (H840A) fused to a RT to directly write new genetic information into the DNA via a pegRNA, enabling a range of modifications, including precise base substitutions, insertions and deletions without the need for DSBs. Each method offers unique editing outcomes, from indel formation with Cas9 nuclease to precise, programmable base edits and structural changes with BEs and prime editors.

Base editing, which harnesses deaminase enzymes to mediate direct nucleotide conversions, has emerged as a pivotal advancement, circumventing the challenges associated with DSB-based gene-editing tools. Base editors (BEs) modify nucleobases by deaminating the amine group of cytosine or adenine, followed by endogenous DNA repair mechanisms that convert cytosine to thymine or adenine to guanine, respectively^11–13^ (Fig. 1). This approach allows precise gene correction without the need for DNA cleavage. Although the base editing field has advanced rapidly, enabling a broader range of nucleotide conversions, current iterations remain restricted to specific base transitions. Moreover, BEs often exhibit off-target modifications, such as bystander editing, where adjacent nucleotides are unintentionally altered, highlighting the need for further refinement to enhance specificity and expand editing capabilities.

To overcome the limitations of both nuclease-based and base-editing technologies, prime editing was developed as a more versatile and precise method for genome modification^14,15^. Prime editors, unlike their predecessors, do not rely on DSBs or donor DNA templates and can introduce a wide range of edits, including all possible base-to-base conversions, small insertions, deletions and even combinations of edits, all without inducing breaks in the DNA. By fusing a reverse transcriptase (RT) to a modified Cas9 nickase (nCas9), prime editors utilize a prime editing guide RNA (pegRNA) to direct the introduction of specific edits at target loci. This innovative approach significantly reduces the risks of unwanted mutations and bystander editing, addressing key limitations of earlier gene-editing platforms. In this Review, we will discuss the recent advancements in prime editing technology, highlight its emerging applications and explore future directions that hold promise for its therapeutic potential and broader applications in genome engineering.

## The architecture and mechanisms of prime editing

Although BEs are a powerful tool for precise nucleotide conversions without inducing DSBs, they have limitations, including small editing windows of four to five nucleotides in the spacer region and dependence on protospacer adjacent motif (PAM) requirements, which restrict their targeting scope^16^. Moreover, BEs can induce off-target mutations in both DNA and RNA, leading to unintended edits due to the deaminase activity of APOBEC and TadA enzymes used in these editors^17–21^. This off-target activity leads to a substantial challenge for therapeutic applications where precision is required. In addition, while efforts are being made to expand BEs beyond C-to-T and A-to-G conversions, they still cannot achieve all desired nucleotide corrections, leaving certain base transitions and transversions unaddressed^22–24^.

Prime editing was developed to overcome these limitations by enabling all types of DNA substitutions, small insertions and deletions. Prime editing is a novel ‘search-and-replace’ genome editing technology that significantly advances the capabilities of the CRISPR system by enabling the introduction of precise edits without the need for DSBs or donor DNA templates^14^. A prime editor consists of a nCas9 (H840A) endonuclease fused to an engineered RT (MMLV RT), programmed with a pegRNA. The pegRNA is a complex molecule that contains both a spacer sequence that identifies the target DNA site and a RT template (RTT) sequence that encodes the desired edit (Fig. 1).

The process begins when the Prime Editor (PE) complex directed by the pegRNA, binds to the target DNA sequence. The nCas9 (H840A) nicks the nontarget strand of DNA, exposing a 3′-hydroxyl group. This exposed end acts as a primer for the RT to extend the DNA using the RTT provided by the pegRNA. This is followed by the formation of a branched intermediate, where the original unedited strand and the newly synthesized edited strand temporarily coexist. To resolve this intermediate, cellular mechanisms first remove the unedited 5′ flap, then ligate the edited 3′ flap to the complementary DNA strand, incorporating the edit into the genome. This approach allows not only all 12 possible base-to-base conversions but also targeted small insertions and deletions, providing a versatile tool for genomic research and potential therapeutic applications. The precision and flexibility of PE represents a substantial improvement over previous genome editing technologies, which often required DSBs and had higher risks of unwanted mutations or complex off-target effects.

## Development versions of prime editors (PE1, PE2 and PE3)

**Fig. 2:**
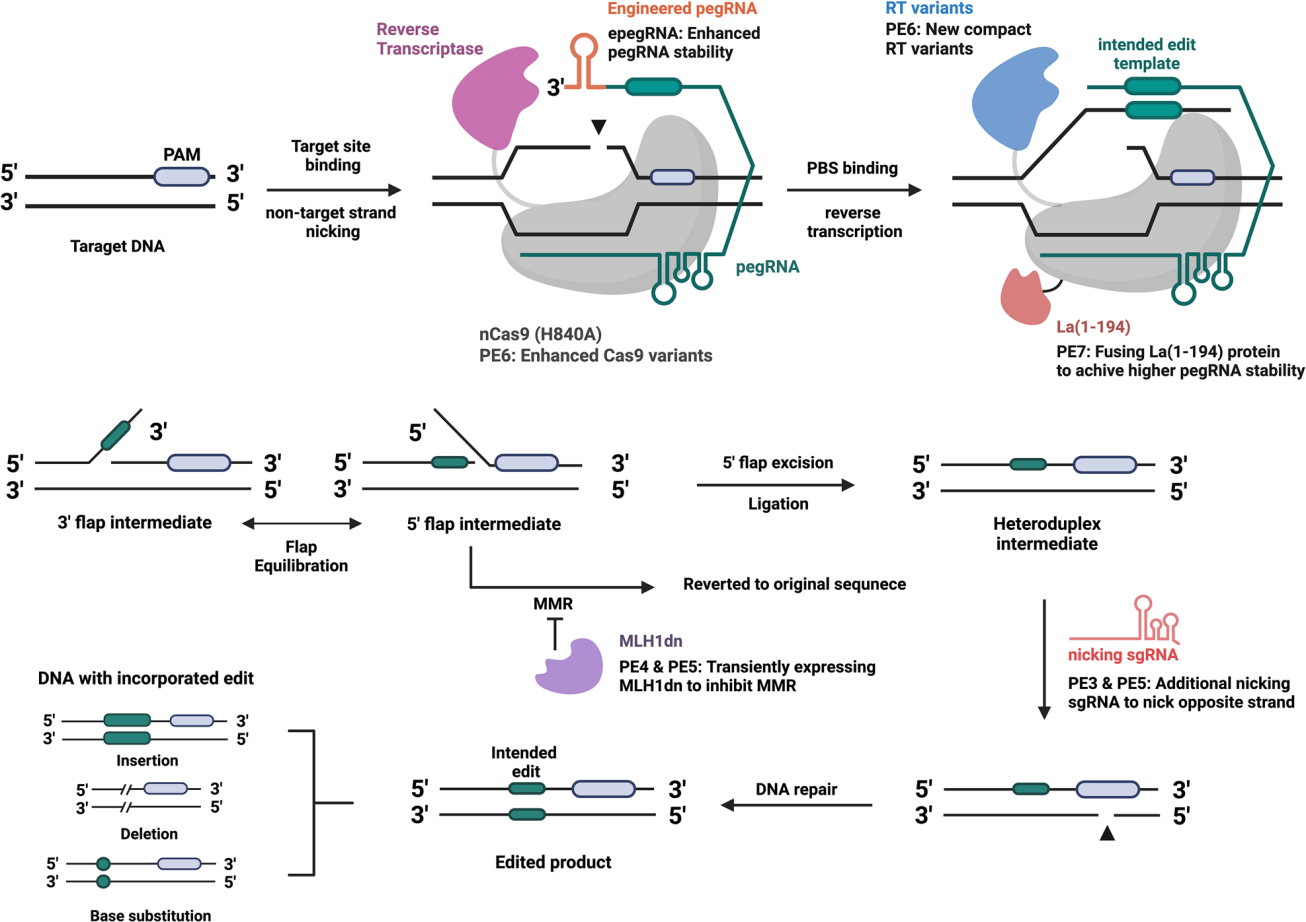
Overview mechanism and evolution of the prime editing system. This figure outlines the mechanism of prime editing and the substantial advancements made in later versions (PE3 to PE7). nCas9 (H840A) initiates the process by introducing a nick on the nontarget strand of DNA, while the attached RT, guided by a pegRNA, synthesizes the desired genetic modification into the DNA. This creates a 3′ flap intermediate, which equilibrates into a 5′ flap, allowing the unedited sequence to be excised and replaced with the intended edit. epegRNAs include additional motifs at the 3′ end to stabilize the pegRNA, leading to higher editing frequencies. PE3 adds an additional nicking sgRNA to the opposite strand to promote efficient repair through the edited strand, resulting in higher editing efficiency. In PE4 and PE5, the inclusion of MLH1dn suppresses the MMR pathway, increasing accuracy and reducing indels. PE6 introduces an optimized, compact RT and enhanced nCas9 (H840A) variants to further improve efficiency. Finally, PE7 incorporates the La(1–194) protein, which stabilizes the pegRNA, leading to further increases in accuracy and efficiency. These improvements collectively demonstrate the progression of prime editing technology, enhancing its precision, stability and applicability in genome engineering.

The development of prime editing began with PE1, which laid the foundational concept of coupling a nCas9 (H840A) with RT to mediate editing. However, the efficiency of PE1 was relatively limited, prompting further innovations to enhance the capability and efficiency for genome editing. PE2 was developed by optimizing the RT fused to nCas9 (H840A), leading to increased fidelity and efficiency of the editing process. Modifications in PE2 included mutations that enhanced the thermostability, processivity and affinity for the RNA–DNA hybrid substrates, which are crucial for efficient and accurate prime editing. These changes resulted in improved editing outcomes, increasing both the range of editable genomic locations and the percentage of successfully edited cells without raising the level of unintended edits or insertions or deletions (indel). Based on the success of PE2, PE3 was introduced to further enhance editing efficiency (Table 1). PE3 incorporates an additional guide RNA that nicks the nonedited DNA strand opposite the pegRNA-guided nick. This additional nicking at opposite strand is designed to encourage the cellular repair machinery to use the newly synthesized edited strand as the template for repairing the nicked complementary strand, thereby increasing the incorporation of the edit into the genome. This system effectively increases the likelihood of successful edits, particularly in challenging genomic contexts or where higher editing efficiency is needed^14^ (Fig. 2).

**Table 1:** Features of prime editor variants.

Type	Cas protein	Components	Editing frequency	pegRNA type	Features	Study
**PE1**	Nickase Cas9 (H840A)	RT (M-MLV RT), pegRNA	~10–20% in HEK293T cells	Linear pegRNA	Initial version of prime editing, demonstrated proof of concept for search-and-replace genome editing	Anzalone et al.^14^
**PE2**	Nickase Cas9 (H840A)	Improved RT (M-MLV RT), enhanced pegRNA design	~20–40% in HEK293T cells	Linear pegRNA	Increased editing efficiency compared with PE1, optimized RT for higher processivity and stability	
**PE3**	Nickase Cas9 (H840A)	RT (M-MLV RT), pegRNA, additional sgRNA for nicking the nonedited strand	~30–50% in HEK293T cells	Linear pegRNA	Introduced dual nicking to enhance editing efficiency, promotes usage of the edited strand as a template for repair	
**PE4**	Nickase Cas9 (H840A)	RT (M-MLV RT), pegRNA, dominant-negative MLH1 (MLH1dn) to inhibit MMR	~50–70% in HEK293T cells	Linear pegRNA	Increased editing efficiency by suppressing MMR pathways, reduced indel formation	Chen et al.^30^
**PE5**	Nickase Cas9 (H840A)	RT (M-MLV RT), pegRNA, additional sgRNA for nicking the nonedited strand, dominant-negative MLH1 (MLH1dn)	~60–80% in HEK293T cells	Linear pegRNA	Enhanced efficiency and precision by inhibiting MMR pathways and using additional sgRNA to nick the nonedited strand	
**PE6**	Nickase Cas9 (H840A)	Modified RT (M-MLV RT, PE6d), new compact RT variants (PE6a, PE6b, PE6c), enhanced Cas9 variants (PE6e, PE6f, PE6g), epegRNAs	~70–90% in HEK293T cells	epegRNA	Variants with compact RT for better delivery, stabilized pegRNAs to reduce degradation	Doman et al.^41^
**PE7**	Nickase Cas9 (H840A)	Modified RT (M-MLV RT), epegRNAs, La(1–194) protein fused to prime editor complex	~80–95% in HEK293T cells	epegRNA with La protein	Improved pegRNA stability and editing efficiency, enhanced editing outcomes in challenging cell types	Yan et al.^42^
**Cas12a PE**	Nickase Cas12a (R1226A)	RT (M-MLV RT)-MCP, circular RNA for reverse transcription-MS2, crRNA for targeting	Up to 40.75% in HEK293T cells	Circular pegRNA (cpegRNA)	Smaller size compared with Cas9-based systems, preferential targeting of T-rich PAMs, enhanced stability and reduced degradation by circular RNA, potential for multiplex editing	Liang et al.^38^
**DPE**	Nickase Cas9 (H840A)	MCP-fused DNA-dependent DNA polymerase (phi29), DNA polymerase editing template (DPET) including MS2 loop	Up to 60% in HEK293T cells	Additional DNA template with gRNA	Uses DNA polymerase instead of RT; separate DNA template is easily synthesized and modifiable for cellular stability	Liu et al.^45^
**CE**	Nickase Cas9 (H840A)	DNA-dependent DNA polymerase, click DNA (clkDNA), HUH endonuclease domain	Up to 25.2% in HCT116 cells	Additional DNA template with gRNA		Ferreira da Silva et al.^46^

This table provides a comparative overview of different prime editing systems, ranging from PE1 to PE7, Cas12a-based prime editors and DNA polymerase-based genome editing tools.

The evolutionary path from PE1 through PE3 illustrates a continuous effort to refine the technology, making it more efficient and robust. Each version has expanded the scope of prime editing, allowing researchers to achieve more precise genetic modifications with fewer byproducts and lower risks of off-target effects, broadening the potential applications of this revolutionary technology in medicine, agriculture and beyond.

## Innovative improvements in prime editing

### Engineering of pegRNA

PE consists of PE protein, a fusion of nCas9 (H840A) and RT, guided by a pegRNA that specifies the target site and contains the desired edit. The original pegRNAs, however, are prone to degradation, reducing editing efficiency. To overcome this limit, structured RNA motifs (evopreQ and mpknot) at the 3′ end of the pegRNA are incorporated, protecting it from degradation^25^. Similarly, independent studies have introduced modifications such as a Zika virus exoribonuclease-resistant RNA motif (xr-pegRNA), a G-quadruplex (G-PE) or a stem–loop aptamer (split prime editor, sPE) to the 3′ extension of pegRNAs, demonstrating comparable improvements in prime editing efficiency in mammalian cells^26–28^. These engineered pegRNAs (epegRNAs) significantly improve editing efficiency by 3–4-fold across multiple human cell lines and primary human fibroblasts, without increasing off-target effects. By stabilizing the 3′ extension, epegRNAs ensure that more prime editor proteins are available for productive editing, reducing the formation of editing-incompetent complexes. Prime editing with epegRNA provides a robust approach to prime editing, enhancing its reliability and precision, with substantial implications for therapeutic applications and disease model development (Fig. 2).

### Engineering of PE proteins for minimizing unwanted indels

PE, which utilizes a fusion of nicakse Cas9 (H840A) and RT, guided by a pegRNA, typically involves creating single-strand breaks instead of DSBs. However, the commonly used nCas9 variant, H840A, can inadvertently generate DSBs, leading to unwanted indels. To address this issue, additional mutations were inserted into nCas9 (H840A), specifically the N863A mutation, which significantly reduced the enzyme’s ability to create DSBs. The modified nCas9 (H840A + N863A) demonstrated a lower frequency of off-target and on-target DSBs, thereby minimizing indel formation. When incorporated into prime editors (PE2 and PE3) and combined with epegRNAs, the nCas9 (H840A + N863A) variant improved the purity of editing outcomes by significantly reducing unwanted indels while maintaining efficient target editing^29^. The potential of using engineered nCas9 to enhance the precision of prime editing makes it a more reliable tool for therapeutic genome editing and other applications requiring high-fidelity genetics.

### sPE system

The substantial size and structural complexity of traditional prime editors have limited their delivery efficiency and therapeutic application, prompting the development of smaller prime editors to overcome these challenges. Attempts to reduce PE size have included using smaller Cas9 orthologs or removing the RNase H domain from the MMLV RT, but these modifications are still not suitable for adeno-associated virus (AAV) delivery^30–33^. The development of sPEs addresses the challenges posed by the large size and complexity of traditional prime editors, which have made delivery and optimization difficult^34^. Unlike previous approaches that require intricate engineering to reassemble the editing components, this new sPE design allows nCas9 and RT to function independently. This separation not only maintains the high precision of genome editing seen in full-length PE3 but also avoids an increase in undesirable indel mutations. In practical applications, the sPE demonstrated its efficacy by successfully editing the β-catenin gene in the mouse liver, leading to tumor formation, and correcting a mutation in a mouse model of type I tyrosinemia using a dual AAV vector system^34^. In addition, the study introduced an innovative approach to splitting pegRNAs into a single guide RNA (sgRNA) and a circular RNA RT template, enhancing their stability and flexibility. This sPE system, which does not rely on inteins, protein–protein affinity modules or sensitive pegRNA extensions, simplifies the construct while preserving efficiency, making it a promising tool for advancing the therapeutic potential of prime editors.

### Overcoming the PAM limitation of PE using Cas variants

PEs, like CRISPR–Cas systems, are constrained by PAM requirements, which limit the range of targetable sites within the genome^35^. To broaden the range of targetable sites, Kweon et al.^36^ developed several engineered spCas9 variants, including VQR, VRQR, VRER, NG, SpG and SpRY, each with expanded PAM recognition capabilities. These variants were integrated into the prime editing system PE2 to create new prime editors that can target a wider array of genomic sites, with prime editing activities reaching up to 51.7%. The PE2-SpRY variant was particularly notable for its ability to target sites with minimal PAM restrictions, enabling edits that were previously inaccessible, such as the BRAF V600E mutation. In addition, the lengths of the primer binding site (PBS) and RT templates were optimized, to maximize editing efficiency. The study also explored the use of the PE3 and PE3b systems to further enhance editing outcomes by introducing additional nicks, which improved the editing efficiency without increasing the rate of unwanted indels.

Furthermore, the use of alternative Cas9 orthologs, such as SaCas9 and cjCas9, has been shown to reduce PE size and diversify PAM requirements. Cas12a, which recognizes T-rich PAMs and functions well in GC-rich regions^37^, has also been used to broaden the application of prime editors^38^. Engineered circular RNA-mediated prime editors (CPEs) based on nickase Cas12a (R1226A)^39^ have been developed. These CPE systems leverage the smaller size of Cas12a and its preference for T-rich PAMs, allowing more versatile and efficient editing (Table 1). The use of circular RNAs enhanced stability and reduced degradation, addressing a major challenge in prime editing^38^. Expanding the versatility of prime editing to overcome traditional PAM limitations makes it a more powerful tool for precise genome editing across a broader range of target sites, with important implications for both research and therapeutic applications.

## Development of PE4 and PE5

Prime editors have evolved through various approaches to enhance their efficiency and precision, with PE4 and PE5 representing substantial advancements by manipulating cellular mismatch repair (MMR) pathways to improve genome editing outcomes. PE4 and PE5 are enhanced versions of the prime editing system that incorporate a strategically engineered suppression of the MMR pathway to increase the efficiency and precision of editing (Table 1). This approach was motivated by findings from pooled CRISPR interference screens that identified the MMR system as a critical modifier of prime editing efficiency. The MMR system, typically involved in the correction of DNA mismatches and small insertion–deletion loops, was found to inhibit prime editing by promoting undesired indel formations and correcting the mismatches introduced during the prime editing process^30,40^.

In the development of PE4 and PE5, a dominant-negative MLH1 (MLH1dn), was used to transiently inhibit the MMR pathway. MLH1dn acts by forming nonfunctional MMR complexes, effectively reducing the fidelity of the MMR mechanism, and allowing the prime-editor-induced mismatches to persist and be incorporated into the genome more reliably. This manipulation has been shown to increase the efficiency of base substitutions, small insertions and deletions by an average of 2.0-fold compared with earlier versions such as PE3, and by 7.7-fold compared with PE2, especially in MMR-proficient cell types (Fig. 2). These systems also involved in understanding the specific interactions between the prime editing machinery and cellular DNA repair mechanisms. The development suggested that MMR typically suppresses prime editing efficiency through its corrective actions on the mismatches formed between the DNA flap, created by the RT activity of the prime editor, and the target DNA strand. By inhibiting MMR, PE4 and PE5 reduce this corrective action, increasing the incorporation of the edited strand into the DNA.

Moreover, the use of PE4 and PE5 has been shown to enhance the ratio of correct edits, a measure of editing precision, by approximately 3.4-fold in various cell types, including HEK293T cells and induced pluripotent stem cells. This enhancement is crucial for therapeutic applications where precision is required to ensure that the intended genetic modifications do not introduce harmful mutations or off-target effects.

PE4 and PE5 represent a major advancement in genome editing by providing a deeper understanding of how cellular DNA repair mechanisms can be leveraged to improve prime editing. By modulating the MMR pathway, these versions significantly enhance the efficiency and accuracy of edits, reducing the formation of unwanted indels. This innovative approach highlights the synergy between genetic engineering and cellular biology, offering promising new strategies for treating genetic diseases that require highly precise and efficient DNA modifications.

## Development of PE6

The prime editor 6 (PE6) system integrates modifications in both the Cas9 protein and RT components, leveraging insights from phage-assisted continuous evolution to refine the efficiency of these enzymes^41^ (Table 1). The adaptations in PE6 are aimed at overcome limitations observed in previous versions of prime editor (such as PE3 and PE5), by addressing issues of size constraints for effective in vivo delivery and the need for higher editing precision without increasing off-target activity. PE6 encompasses a range of specialized variants (PE6a to PE6g), each incorporating a range of modifications to optimize different aspects of the prime editing process, such as improved delivery mechanisms, increased editing efficiency and reduced off-target effects (Fig. 2).

PE6a and PE6b feature compact versions of RT that are smaller in size, making them more suitable for delivery vectors with size limitations, such as AAVs. These compact RTs are developed to maintain high editing efficiency despite their reduced size, which is crucial for therapeutic applications where delivery efficiency is paramount. PE6c to PE6f focus on enhancing the RT and Cas9 components to improve overall editing efficiency and specificity. These variants have been engineered to perform better under different cellular conditions, and in different cell types, including those that are typically challenging to edit. These achieved mutations enhance the fidelity and activity of the RT and Cas9 enzymes, ensuring that the edits not only are precise but also minimize unintended edits elsewhere in the genome.

Moreover, PE6 introduces modifications that enhance the performance of the prime editing system in therapeutically relevant cell types, such as T cells and other primary cells, which are crucial for the application of prime editing in gene therapy. The specific adjustments in PE6 variants facilitate the installation of long edits and complex genomic rearrangements with higher efficiency and lower toxicity compared with earlier prime editor versions. PE6 significantly broadens the utility of PE for both research and therapeutic applications. This progression not only exemplifies the rapid advancements in genome editing technologies but also highlights the potential for these tools to contribute to precision medicine and genetic research.

## Development of PE7

PE7 represents a groundbreaking advancement in the prime editing field, incorporating novel strategies to enhance its efficiency and applicability (Table 1). PE7 incorporates the N-terminal domain of the La protein (1–194) directly fused to the prime editor complex. This modification uses its ability to bind and stabilize polyU sequences at the 3′ ends of RNA molecules. The La protein is known for its role in protecting nascent RNA polymerase III transcripts from exonucleolytic degradation^42–44^, and its integration into the prime editing system aims to stabilize the pegRNAs, therefore enhancing the efficiency of the prime editing process (Fig. 2).

The development of PE7 was motivated by the discovery, through comprehensive genomic screens, that the La protein significantly enhances prime editing outcomes across various cell types and genomic targets. These screens revealed that La improves the stability of pegRNAs in the cellular environment, an important factor for susceptibility of pegRNAs to degradation. By fusing La(1–194) to the prime editor, the enhanced stability of pegRNAs directly leads to increased editing efficiency. This fusion strategy ensures that the protective effects of La are localized precisely at the site of editing, thereby minimizing off-target effects and maximizing the fidelity and efficiency of the edits.

PE7 has been shown to significantly outperform earlier versions of prime editors, such as PEmax, particularly in cells where prime editing has traditionally been less efficient or more challenging. The enhancement is largely attributed to the improved stability and function of the pegRNAs, which are less likely to degrade and more likely to successfully mediate the desired genomic modifications.

By harnessing the natural properties of the La protein to enhance the stability and function of pegRNAs as well as precise and reliable genome editing outcomes, PE7 represents a substantial step forward in the field of genome editing for research and therapy. This advancement not only states the importance of understanding and integrating cellular and molecular dynamics into genome editing strategies but also highlights the potential for innovative approaches to overcome the limitations of existing technologies.

## DNA-dependent polymerase-based genome editing

Recently, two research groups reported gene-editing technologies using DNA-dependent polymerase, introducing DNA polymerase editors (DPEs) and click editors (CEs)^45,46^ (Table 1). These tools are an innovative genome editing approach that, like prime editing, avoids the need for DSBs, thereby reducing the risks associated with traditional CRISPR-based systems. Whereas prime editing uses a RT to introduce specific edits guided by a pegRNA, DPEs use a similar strategy of precision by utilizing DNA-dependent polymerases. This system provides a novel mechanism for targeted base editing, aiming for higher accuracy and efficiency. CEs use a similar approach but incorporate additional chemical modification steps to further enhance the precision of genome editing. DNA polymerases and HUH endonucleases work together to install edits at targeted genomic sites through a click-like bioconjugation process, where clkDNAs, single-stranded DNA templates, are tethered and incorporated into the genome with high precision. Both technologies represent substantial advancements in genome editing by allowing a wide range of genetic modifications, including substitutions, insertions and deletions, with minimal indel formation, making them powerful tools for various biological applications where traditional methods fall short.

## Application of prime editing tools

### Prime editing for large gene modifications

**Fig. 3:**
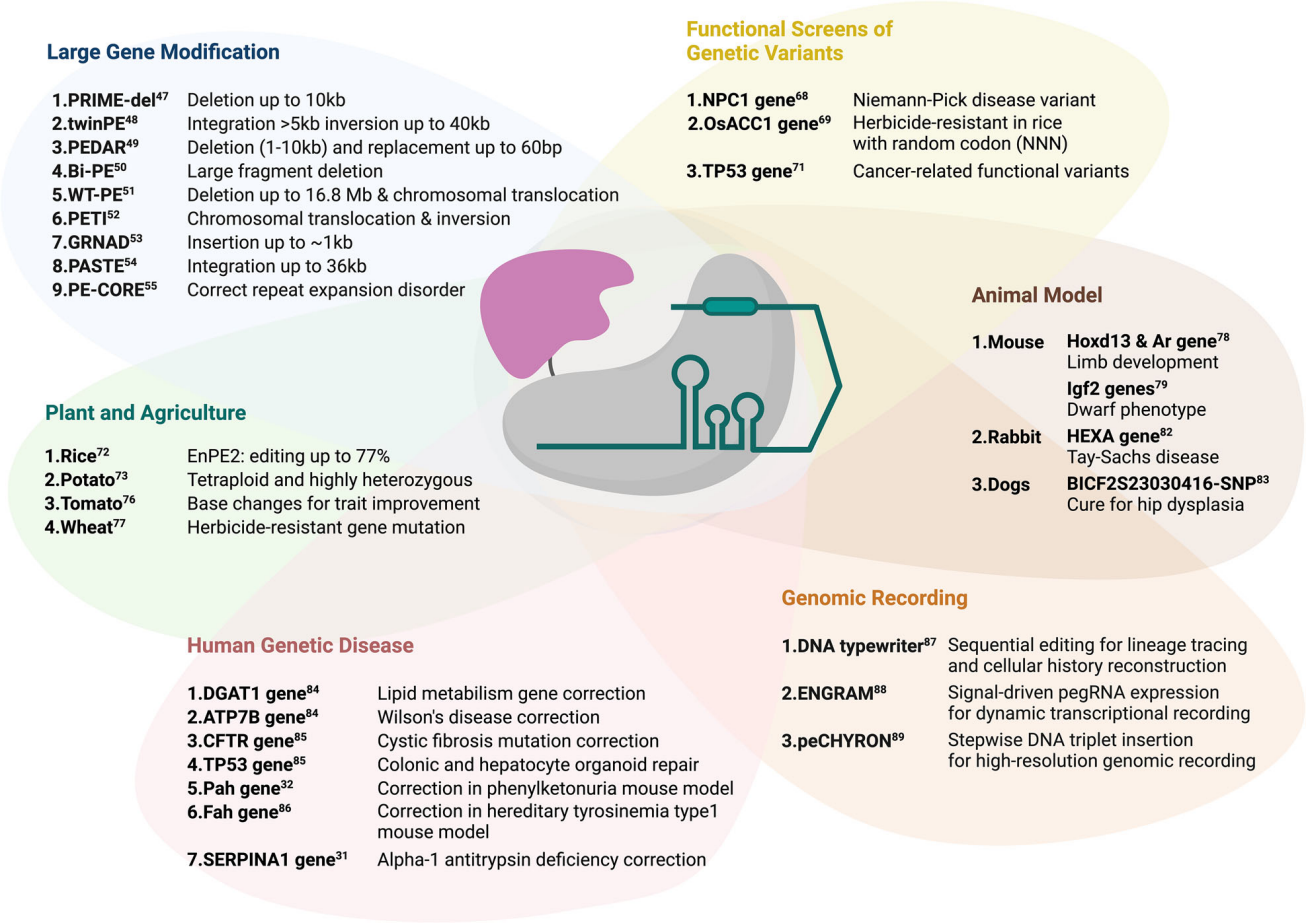
Overview of applications of prime editing. This figure illustrates the diverse applications of PE across large gene modifications, functional screens of genetic variants, plant and agriculture, animal models, human genetic diseases and genomic recording. PE enables precise genome modifications, including deletions, insertions, inversions and translocations. It facilitates the study of genetic variants, the development of animal models for disease research, targeted genetic improvements in crops and therapeutic correction of hereditary disorders. These applications highlight the broad utility and precision of prime editing in scientific research, therapeutic advancements and agricultural innovation.

It has been reported that using a prime editor protein in conjunction with paired pegRNAs can expand the range of potential applications. This approach not only increases the efficiency of introducing desired mutations at target sites but also enables precise, large-scale genetic modifications (Fig. 3). Notable techniques in this area include PRIME-del^47^ and twinPE^48^, which allow deletions of up to 10 kb and insertions of genes up to 250 bp. In addition, substituting nCas9 (H840A) in PE with wild-type Cas9 can induce DSBs at target sites during prime editing, potentially enhancing editing efficiency and enabling larger genetic modifications. Techniques such as PEDAR^49^, Bi-PE^50^ and WT-PE^51^ have demonstrated the capability for large deletions and insertions, while PETI^52^ has shown the feasibility of inducing chromosomal translocations and inversions. Expanding its versatility, prime editing has also been adapted for large gene insertions, with methods such as GRAND^53^, which utilizes paired PE and long pegRNAs for direct insertions, and twinPE with integrase^48^ or PASTE^54^, which incorporate recombinases or transposases to improve efficiency. Furthermore, PE-CORE^55^ has been introduced as a strategy to precisely delete pathogenic repeat sequences, offering potential therapeutic applications for repeat expansion disorders. These advancements further extend the capability of prime editing for precise large-scale genome modifications, broadening its impact on complex genome engineering.

### Functional screens of genetic variants

Advancements in CRISPR–Cas9 technology have significantly enhanced functional genetic screens. Nuclease-based CRISPR approaches initially enabled efficient knockout screens^4,56^, systematically inactivating genes to reveal their roles, while CRISPR interference and CRISPR activation systems allowed screens based on gene repression or activation^57–59^, providing insights into gene regulation without altering DNA sequences. To investigate patient-specific genetic variants, saturation genome editing (SGE) was developed, utilizing homology-directed repair (HDR) to introduce precise nucleotide changes, as applied in genes such as *BRCA1* (refs. ^60–62^). However, the reliance of SGE on low-efficiency HDR has limited its use, leading to the development of BE-based screens that bypass DSBs for more efficient and precise nucleotide changes^63–67^. More recently, prime editing has shown promise in functional screens of genetic variants^68–71^, offering key advantages over both SGE and BEs (Fig. 3). Cohn and colleagues used prime editing to evaluate NPC1 variants of unknown significance linked to Niemann–Pick disease type C1, as a proof of concept^68^. Wei and colleagues applied prime editing with pegRNAs containing random codons (NNN) to mutagenize *OsACC1* in rice, identifying herbicide-resistant variants via a random-PE approach. In addition, successful genetic screening of TP53 and DNA noncoding elements has also been reported, further demonstrating PE’s versatility^70,71^. PE enables a broad range of modifications, including all base-to-base conversions and small insertions and deletions, without inducing DSBs. Using pegRNA to guide edits and avoid HDR dependency, PE provides high accuracy across various genomic contexts, including nondividing cells, where HDR is inefficient. By significantly reducing unintended mutations, PE represents a powerful tool for disease modeling and therapeutic research, addressing complex genetic variations with a high degree of precision.

### Prime editing in plants and agriculture

Prime editing is emerging as a transformative tool in plant genome engineering, enabling precise genetic modifications across various crop species^72–76^ (Fig. 3). Studies have demonstrated its potential to revolutionize crop improvement and functional genomics, with notable success in rice through the enhanced prime editor 2 system (enpPE2), achieving editing efficiencies up to 77% and facilitating key agronomic gene modifications for improved yield and disease resistance^72^. Prime editing has also been applied to dicotyledonous plants such as tomato, allowing specific base changes that enhance trait development and functional studies^76^. Beyond single-species applications, prime editing has been used across multiple plants, such as wheat, to introduce herbicide-resistant mutations, showcasing its potential to address agricultural challenges^77^. The ability to target multiple loci simultaneously makes it particularly valuable for polyploid crops, broadening its use in plant breeding. As prime editing technology advances, it holds promise to revolutionize crop improvement and sustainable agriculture, contributing to the development of varieties with enhanced yield, nutritional content and environmental resilience.

### Prime editing in animal models

Prime editing has emerged as a transformative tool for generating animal models that closely mimic human genetic diseases, offering crucial insights into disease mechanisms and potential therapies. By enabling precise genetic modifications without DSBs, prime editing is ideal for creating accurate animal models (Fig. 3). In mice, studies have shown its effectiveness in replicating human conditions, such as in models with mutations in the *Hoxd13* and *Ar* genes, and enhanced versions of the editor have allowed targeted mutagenesis in embryos for refined model creation^78–81^. For instance, Liu et al.^78^ used prime editing to introduce specific mutations in the Hoxd13 and Ar genes, creating mouse models that closely mimic these human disease-related mutations. In addition, Park et al.^79^ utilized an enhanced version of the prime editor to achieve targeted mutagenesis in mouse cells and embryos, successfully creating precise genetic modifications that are essential for developing sophisticated mouse models. These enhancements in prime editing highlight its growing potential for creating accurate and efficient animal models. Prime editing has also been pivotal in developing rabbit models for diseases that are challenging to study in rodents. Qian et al.^82^ used prime editing to create a Tay–Sachs disease model by introducing a mutation in the HEXA gene. This approach allowed the generation of rabbits exhibiting human-like disease phenotypes, which are essential for understanding the disease and testing potential treatments. In dogs, prime editing has been used to correct genetic mutations associated with hereditary conditions. Kim et al.^83^ applied prime editing to correct a mutation linked to hip dysplasia in Labrador retrievers, successfully generating gene-corrected dogs. This application of prime editing has produced a valuable model for studying the disease and developing interventions that could also benefit human medicine. These examples highlight the power of prime editing in generating precise animal models for human genetic diseases, demonstrating its potential to advance research and therapeutic development significantly.

### Human genetic disease therapy

Prime editors have demonstrated considerable potential for correcting mutations linked to various human genetic diseases (Fig. 3). In organoid models, prime editing has been successfully applied to repair the CFTR-F508del mutation, which causes cystic fibrosis, as well as correct mutations associated with DGAT1 deficiency and Wilson’s disease. In addition, prime editing has been applied in organoids to model and correct cancer-associated mutations, such as the TP53 mutations in colonic and hepatocyte organoids, underscoring its potential for both disease modeling and therapeutic correction^84,85^. Beyond organoids, prime editing has shown promise in in vivo models, achieving genomic correction efficiencies of 11.1% in a Pah model of phenylketonuria^32^ and 11.5% in a mouse model of hereditary tyrosinaemia^86^, targeting liver tissue. Further research highlights the potential for prime editors to correct the SERPINA1 E342K mutation responsible for α-1 antitrypsin deficiency, achieving up to 15.8% correction in hepatocytes^31^. These examples underscore the potential of prime editing to treat a wide range of genetic disorders, paving the way for future therapeutic applications.

### Genomic recording: capturing cellular events in DNA

DNA serves as a natural medium for in vivo molecular recording, and recent advances have expanded its utility for capturing complex biological events with high resolution (Fig. 3). DNA Typewriter^87^ introduces a sequential genome editing system that enables the ordered recording of biological signals through the progressive insertion of distinct symbols into a genomic ‘DNA Tape’ via prime editing. This system enables precise lineage tracing and reconstruction of cellular histories over multiple generations. Building on this, ENGRAM^88^ utilizes signal-responsive *cis*-regulatory elements to drive pegRNA expression, stably recording transcriptional activity and dynamic signaling events such as WNT and NF-κB pathways. Meanwhile, peCHYRON^89^ enhances temporal resolution by inserting distinct DNA triplets at each recording step, enabling durable and multiplexed encoding of cellular experiences. Together, these genomic recording technologies offer scalable, noninvasive methods to track dynamic biological processes, with applications in lineage tracing, developmental biology and disease modeling.

## Future challenges

### Efficiency and specificity

Despite substantial advancements, achieving high efficiency and specificity across diverse cell types and organisms remains a challenge^14^. Prime editing efficiency can vary significantly depending on the target site and the cellular context, necessitating further optimization to ensure consistent and robust performance. In particular, in vivo editing efficiency using prime editors has not yet reached the high levels seen with other genome editing tools. Prime editors have consistently demonstrated genomic correction rates below 10%, with specific examples including 6.4% correction in RPE tissue derived from a model of Leber congenital amaurosis and 1.71% correction in the Dnmt1 gene^90^ In terms of specificity, key future challenges for prime editing will be off-target effects, which are unintended modifications at genomic sites other than the intended target. Although PE is considered more precise than traditional CRISPR–Cas9 systems, studies have shown that off-target activity can still occur^91–93^, particularly in regions with sequence similarity to the target site, potentially leading to genomic instability or unwanted mutations.

### Delivery methods

The effective delivery of prime editors to target cells and tissues in vivo is a substantial hurdle due to the combination of nCas9 (H840A) and RT, along with the pegRNA, which complicates their delivery into cells. Current gene delivery systems, such as AAVs, are limited by their small cargo capacity (about 4.7 kb), making it difficult to fit the entire prime editor system into a single AAV vector. This has led to the use of dual-AAV systems, where the prime editor is split between two vectors and reassembled in the cell. However, dual-AAV delivery systems face challenges, including reduced editing efficiency due to incomplete reassembly and lower expression levels. Furthermore, the delivery of PE to specific tissues, especially in therapeutic settings, presents further hurdles. Developing more efficient delivery systems, including nonviral methods, nanoparticles^94^ and virus-like particles^95^, is essential for therapeutic applications. Nonviral methods such as nanoparticles or lipid-based delivery offer an alternative but are still in the experimental phase, requiring further optimization for tissue specificity and efficient cargo protection. The development of more compact prime editors, engineered for size reduction, or new delivery platforms that can handle the large size of PE components will be critical for advancing the application of prime editing in clinical therapies. Overcoming these challenges is essential to ensure the efficient, safe and targeted delivery of prime editors for therapeutic use.

### Miniature prime editor

Current prime editors, which combine the nCas9 (H840A) and RT, are too large to be easily packaged into AAV vectors, the most used delivery vehicles in gene therapy. This size limitation significantly hampers the potential of prime editing for therapeutic use. To address this, researchers are exploring the potential of smaller RNA-guided endonucleases, such as TnpB and IscB, which offer a promising solution. These proteins are much smaller than Cas9, making them attractive candidates for use in genome editing where delivery size is a critical factor. TnpB and IscB have been shown to function as a programmable RNA-guided DNA endonuclease, guided by a short RNA called reRNA or ωRNA derived from the transposon itself. These systems are not only compact but also versatile, as TnpB and IscB can be reprogrammed to target specific DNA sequences, like Cas9, but with a footprint that is approximately half the size^96–98^. This smaller size makes these proteins more suitable for packaging into AAV vectors, potentially overcoming one of the most substantial barriers to the widespread therapeutic application of prime editing. Its compact nature suggests it could be engineered into a new form of prime editor that retains the precision of current systems but with a reduced size. This could enable more efficient in vivo delivery using AAVs or other viral vectors, expanding the therapeutic potential of prime editing.

The development of prime editors based on TnpB and IscB will thus represent a substantial advancement in the field, addressing the current limitations related to size and delivery, and paving the way for more practical and widespread applications of prime editing in clinical settings.

## Conclusion

Prime editing has become a transformative tool in genome engineering, offering enhanced precision and versatility over traditional CRISPR-based methods. Since its introduction, successive versions (PE1 to PE7) have integrated key advancements, including improved RT activity, optimized pegRNA designs and stabilizing elements such as the La(1–194) protein, expanding its range for precise base substitutions, insertions, deletions and even large-scale genomic rearrangements without DSBs. These improvements have broadened the applications of prime editing, from disease modeling to therapeutic corrections for human genetic diseases, highlighting its potential for research and clinical translation.

Nevertheless, challenges remain, particularly in efficiently delivering prime editing components in vivo, given delivery constraints such as AAVs. Effective, tissue-specific delivery methods and further refinements to minimize off-target effects and immune responses to Cas9 and RT proteins are essential for therapeutic use. Moving forward, continued advancements in prime editing systems and delivery platforms will unlock the full potential of this technology, positioning it as a versatile and precise tool for treating genetic disorders and expanding its utility to agriculture, synthetic biology and biotechnology. With ongoing research, prime editing is set to become a cornerstone in next-generation genome engineering for scientific discovery and therapeutic innovation.
